# Effects of fluid shear stress on expression of focal adhesion kinase in MG-63 human osteoblast-like cells on different surface modification of titanium

**DOI:** 10.1080/21655979.2021.1962686

**Published:** 2021-08-10

**Authors:** Xin Lei, Qiong Liu, Shiyi Li, Zhaoqiang Zhang, Xiaoyu Yang

**Affiliations:** a Department of Stomatology, Shenzhen Longhua District Central Hospital, Shenzhen, China; b Stomatological Hospital, Southern Medical University, Guangzhou Guangdong, China

**Keywords:** Titanium surface, fluid shear stress, focal adhesion kinase, cell proliferation

## Abstract

This study aimed to investigate the effect of fluid shear stress (FSS) on cell proliferation and expression of focal adhesion kinase (FAK) in MG-63 cells on different modified titanium surfaces. MG63 cells were cultured on three different surfaces: glass slide, polished treatment (PT) titanium surface and sandblasted/acid-etched surfaces (SLA) titanium surface. The surface topography and roughness were evaluated by scanning electron microscopy (SEM) and atomic force microscopy (AFM), respectively. The cells were subjected to FSS, and the cell appearance before and after the stress was evaluated. MTT assay was applied to estimate cell proliferation. The mRNA and protein levels of FAK were determined by qRT-PCR and western blotting. Titanium plates demonstrated different surface microtopography. Parameter Ra values of SLA group were around 3.4 µm, which was higher than PT group. Exposure to the FSS of 12 dynes/cm^2^ significantly induced positive upregulation of cellular proliferation and the expression of FAK, which were directly correlated with the duration of exposure and surface. Cells in SLA group were able to endurance the longtime of FSS, especially under the FSS of 16 dynes/cm^2^. SLA surface had a positive influence on the expression of FAK. Different surface modifications created different microtopography of titanium plates. Cell proliferation and the mRNA and protein expression of FAK were stimulated by FSS and regulated by a marked synergistic effect of surface topography and the level and duration of FSS.

## Background

Bone integration is critical to the success of an implant. The morphology of an implant surface, such as microtopography and roughness, has been proved to play key roles in successful osseointegration [1]. Titanium implants are commonly utilized in dentistry because of their excellent biocompatibility and adaptability to plastic surface structure [2]. Numerous techniques have been developed to modify the topography of titanium implants. Clinical studies have shown that the pre-loading integration success rate of acid-etched implants is significantly higher than of machined smooth implants [2,3]. The surface with micron-scale roughness, typified by SLA, has significantly positive influence on osteoblast cells [1,4].

The long-term success of dental implants is affected by several factors, including implant biomechanics, distribution of load at the bone–implant interface, and stress transfer to the bone [3,5]. Mechanical compression of bone tissue can induce fluid flow within the perilacunar/canalicular network, contributing to the changes in fluid shear stress (FSS) and hydrostatic pressure sensed by osteocytes [6]. Osteocytes are known to be particularly sensitive to the FSS [6,7]. The physiological level of FSS has been calculated at 0.8–3.0 Pa via applying Biot’s poroelastic media theory to models of the lacunar-canalicular porosity [8,9]. Previously, various studies have investigated the effects of FSS on osteoblasts, but the effects of FSS on osteoblasts on the bone–implants surface are still poorly reported.

Integrin, serving as a transmembrane linker between cell-extracellular matrix (ECM) and cytoskeleton components, transduces biochemical signals to regulate cellular biological functions [10,11]. Whereas, as a result of lacking intrinsic enzymatic activity in integrin cytoplasmic domains, the transmission of the signal is involved in the mediation of cytoplasmic tyrosine kinases [12,13]. Moreover, FAK is a nonreceptor tyrosine kinase found in focal adhesions, plays a critical role in the integrin-mediated signal transduction pathways and links to downstream signaling, regulating activation of Src, Grb2-SOS and MEK [14,15]. Recent studies have provided direct evidence for the involvement of FAK in cellular migration, adhesion, proliferation and apoptosis, but its role in the mechanotransduction on the implant–bone interface has not been examined [16,17]. The present study aimed to investigate the effect of FSS on the cell proliferation and expression of FAK in MG-63 cells on different surface modifications of polished treatment (PT) ritanium surface and sandblasted/acid-etched surfaces (SLA) ritanium surface.

## Methods

### Cell culture

MG63 osteoblast-like cells were purchased from ATCC (CRL-1427, Manassas, VA, USA) and cultured in Dulbecco’s modified Eagle medium (DMEM) containing 10% fetal bovine serum (FBS) and 1% penicillin/streptomycin at 37°C in an atmosphere of 5% CO_2_ and 100% humidity. Cell culture medium (DMEM) and FBS were purchased from GIBCO (Thermo Fisher Scientific, Waltham, MA). The media were exchanged every 48–72 h. When the cells reached 70–80% confluence, they were passaged using trypsin digestion. After 3–4 days of culture, the cells were applied with FSS.

### Cell grouping

MG63 cells were cultured on three different surfaces: glass slide, PT titanium surface and SLA titanium surface.

A pure 1 mm-thick titanium (ShanXi, China) sheet of grade 1 was cut into 75 mm × 25 mm titanium pieces. For PT group, the surface was ground sequentially using waterproof abrasive paper from 360 to 1200. All the samples were ultrasonically washed in pure acetone, absolute alcohol and deionized water for 15 min. For SLA group, the SLA surface was prepared by coarse grit-blasting the PT surface with 0.3–0.8 mm Al_2_O_3_ powder until the surface becomes a uniform gray. The samples were washed in a mixture (1:1:2 by volume) of 98% H_2_SO_4_, 37% HCl, and deionized water at 70°C in Horizontal Shaking BathChrom Technology (USA) for 30 min. Then, samples were washed in 6 M NaOH at 70°C in Horizontal Shaking BathChromTech for 5 h. All the samples were ultrasonically washed in pure acetone, absolute alcohol and deionized water for 15 min, dried in a desiccator, and sterilized.

### Surface roughness characterization

Surface morphology was examined by scanning electron microscopy (SEM) and atomic force microscopy (AFM) [18].

### Flow system

The FSS loading device in this experiment is improved based on the flow chamber system designed by Hochmuch et al. [19]. The fluid loading system is composed of peristaltic pump, silicone tube, liquid storage bottle, buffer bottle and flat flow chamber. A circulation flow system was utilized to impose FSS on cultured MG63 at 37°C. A parallel plate flow chamber was connected to two reservoirs, each containing 100 ml warm medium. A peristaltic pump (GlycoTech, USA) returned medium to maintain a constant level. The samples with cells for flow experiments were placed in the parallel plate flow chamber. The shear stress was calculated (in dyn/cm^2^) from the volumetric flow rate, using the equation τ = 6ηQ/wh^2^; τ, FSS(dynes/cm^2^), η, viscosity of the media (taken to be 0.86 × 10^−2^ dynes. s/cm^2^); h, channel height (0.3 mm); w, channel width (2.5 cm); and Q, volumetric flow rate (cm^3^/s).

### MTT assay

MTT assay was utilized to evaluate the proliferation of MG-63 [20]. Cells were collected at 0, 0.5 h, 1 h, 2 h, 4 h and 8 h after FSS (8 dynes/cm^2^, 12 dynes/cm^2^ and 16 dynes/cm^2^) stimulation. 3 ml DMEM and 120 μl of MTT dye solution were added to each sample and the cells were cultured for 4 h at 37°C in a CO_2_ incubator. Also, 1260 μl of dimethylsulfoxide (DMSO) was added to stop the reaction. After half an hour of culture, 150 μl of the solution was then transferred to 96-well. The absorbance was measured at a wavelength of 490 nm using an ELISA plate reader (Bio-Rad, USA) and calculated by OD sample/OD initial.

q*RT-PCR*

Cells were collected at 0, 0.5 h, 1 h, 2 h, 4 h and 8 h after FSS (8 dynes/cm^2^, 12 dynes/cm^2^ and 16 dynes/cm^2^) stimulation. Total RNA was isolated from the cultured cells using Trizol reagent (Gibco BRL Life Technologies) and. AxyPrep Multisource Total RNA Midiprep Kit (AXYGEN, USA) was used for the extraction of total RNA according to the manufacturer’s protocol. Reverse transcription and quantified PCR was performed using TaKaRa One Step RNA PCR Kit (AMV) (TaKaRa, Dalian, China) according to the manufacturer’s recommendation for the measurement of FAK levels. Glyceraldehyde-3-phosphate dehydrogenase (GAPDH) was identified as the housekeeping gene. The expression of target genes was calculated using 2^−ΔΔCt^ method. The primer sequences were as follows (FAK, F: 5ʹ-ACATTATTGGCCACTGTGGATGAG-3ʹ; R: 5ʹ-GGCCAGTTTCATCTTGTTGATGAG-3ʹ; GAPDH, F: 5ʹ-GCACCGTCAA GGCTGAGAAC-3ʹ, R: 5ʹ-TGGTGAAGACGCCAGTGGA-3ʹ).

### Western blot analysis

Cell or tissue (50 µg) were lysed in RIPA buffer containing 1× PBS, 1% NP-40, 0.1% sodium dodecyl sulfate (SDS), 5 mM EDTA, 0.5% sodium deoxycholate and 1 mM sodium orthovanadate and protease inhibitors. Protein expression levels were assessed by immunoblot analysis. The separated proteins were transferred to PVDF membranes by electrotransfer. Membranes were probed by a rabbit polyclonal FAK, P-FAK (1:1000; CST, USA). Rabbit anti-β-actin (1:500; Santa Cruz, CA) antibody served as an internal control. The protein levels were detected using Image Quant (Molecular Dynamics, Chicago IL) software.

### Statistical analysis

The statistical data were presented as mean ± SD. The level of variance between groups was found to be similar. Statistical analyses were performed by analysis of variance (ANOVA) with Tukey’s post-hoc multiple comparison tests. The differences were considered to be significant at a level of *P* < 0.05.

## Results

### Surface characterization

**Figure 1.**
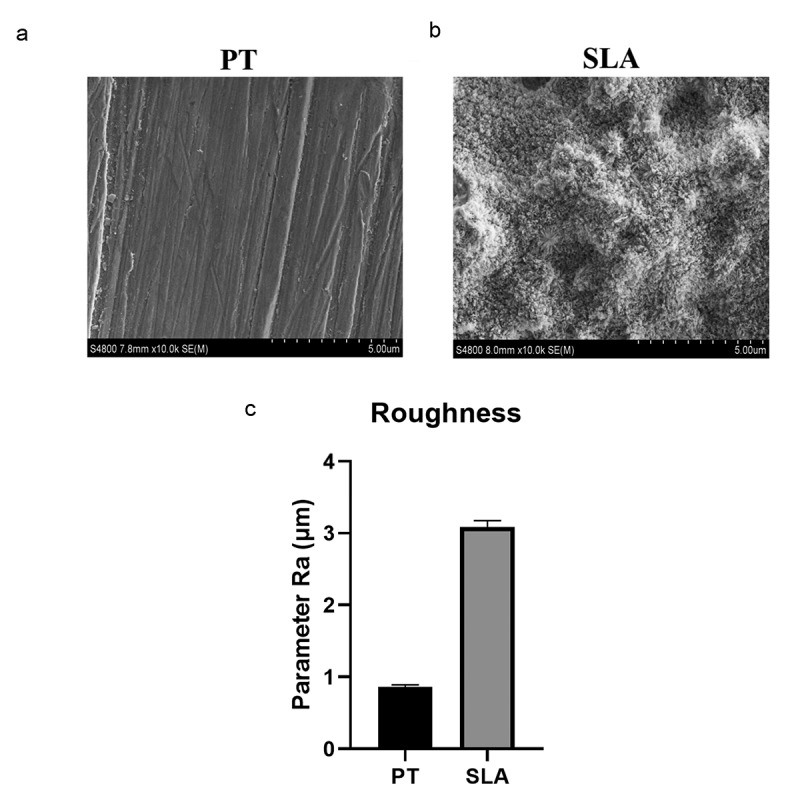
The surface characterization of different titanium. The SEM picture of PT group (a) and SLA group (b), and the mean values of the different titanium surfaces (c)

After different treatments, the surface microtopography of the Titanium plates was evaluated via SEM and AFM. PT group was characterized by mechanical grooves with the same direction (Figure 1a). SLA group had a multiparous microtopography with the diameter of about 5–10 µm (Figure 1b). Parameter Ra value of SLA group was around 3.4 μm, which was higher than PT group (Figure 1c). Titanium plates demonstrated different surface microtopography.

### The proliferation of MG-63 on different implant–bone interface

**Figure 2.**
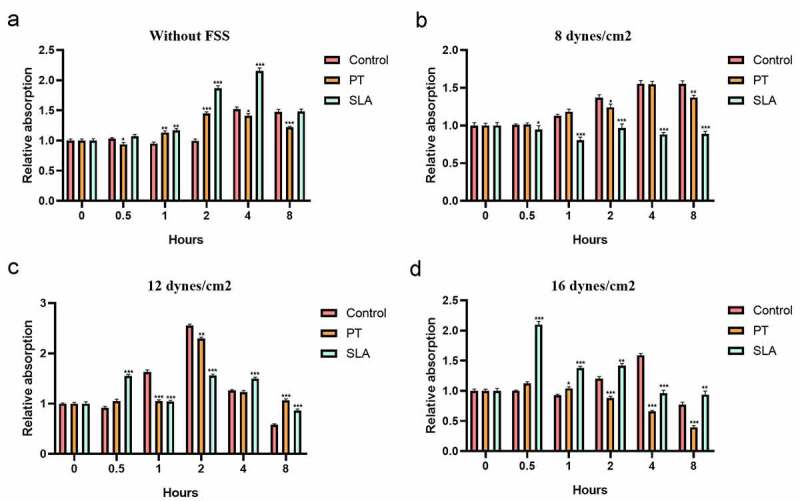
Effect of FSS on the proliferation of MG63 on different titanium implant. Cells were cultured in glass slide (control group), polished treatment titanium surface (PT group), and sandblasted/acid-etched surfaces titanium surface (SLA group). Cells were under static condition (a) and exposed to FSS (8 dynes/cm^2^ (b), 12 dynes/cm^2^ (c), 16 dynes/cm^2^ (d)) for the indicated times. *P < 0.05, **P < 0.01, ***P < 0.001

Then we investigated the effects of various levels and duration of FSS on the proliferation of MG-63 cells on implant-bone interface. Without FSS, the cell proliferation was improved in the SLA group (Figure 2a). Previous studies have reported FSS activated the proliferation of human mesenchymal stem cells or increased the osteogenic differentiation ability [21]. Exposure to the FSS of 8 dynes/cm^2^ failed to upregulate cellular proliferation and the trend was much stabler in cells on SLA than on PT (Figure 2b). Exposure to the FSS of 12 dynes/cm^2^ significantly reduced upregulation of cellular proliferation, which directly correlated with the duration of exposure and surface (Figure 2c). Distinctly, the proliferation of MG63 cultured on SLA group was remarked upregulated within 0.5 h (*P* < 0.001) while MG63 cultured on other surfaces was downregulated (Figure 2c, *P* < 0.001). It implied that the cells cultured on SLA group were more sensitive than other groups. A maximum level was reached within 2 h (*P* < 0.001). Nevertheless, after 8 h shearing treatment, the cell proliferation was inhibited, which was lower than the static level (Figure 2c, *P* < 0.001), even lower than initial level. Whereas the level of SLA group was much stabler than PT group (Figure 2c). It suggested that SLA group might have positive influences on osteoclasts proliferation. Exposure to the FSS of 16 dynes/cm^2^ had significant effects on cellular proliferation, which also directly correlated with the duration of exposure and surface (Figure 2d). Maximum level of cell viability was reached within 0.5 h (Figure 2d, *P* < 0.001). With the duration increases, the cell proliferation was inhibited (Figure 2d). Amazingly, although the levels of the SLA cell viability at 4 h and 8 h was lower than the static, they had no significant difference with the initial level (Figure 2d, *P* > 0.01). It suggested that the multiparous microtopography characterized by SLA may disperse the force so that the cells can endurance under overloading conditions.

### The FAK gene expression in different titanium implant

**Figure 3.**
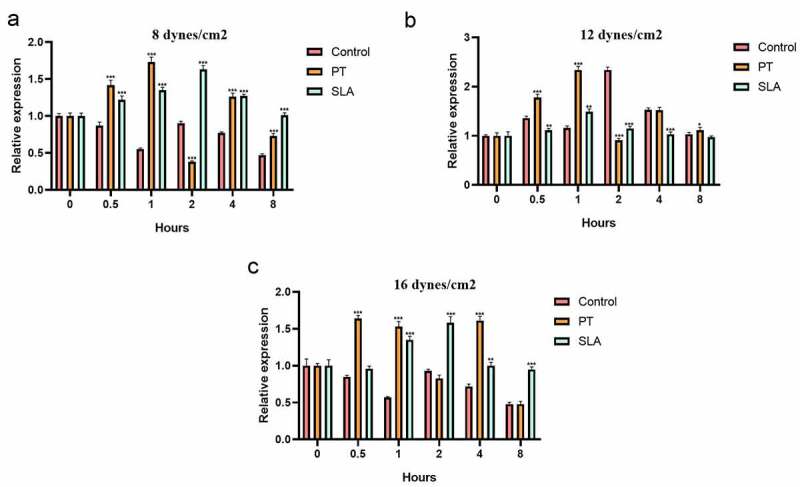
Effect of FSS on the FAK gene expression of MG63 on different titanium implant. Cells were exposed to FSS (8 dynes/cm^2^ (a), 12 dynes/cm^2^ (b), 16 dynes/cm^2^ (c)). *P < 0.05, **P < 0.01, ***P < 0.001

The effects of various levels and duration of FSS on the FAK mRNA expression in MG-63 cells were evaluated. Previous studies have reported FSS induced a rapid and transient activation of FAK [22,23]. Our results showed that exposure to the FSS of 12 dynes/cm^2^ had positive influence on the expression of FAK mRNA, and SLA surface also upregulated the FAK mRNA synergistically which directly correlated with the level and duration of exposure (Figure 3). On glass side group, application of the FSS (8 and 16 dynes/cm^2^) caused sustained decreases of FAK (Figure 3(a–c), *P* < 0.05), and a minimum level was reached at 8 h (Figure 3(a-c) *P* < 0.001). Exposed to 12 dynes/cm^2^, the FAK mRNA level of control group was upregulated and got down at 1 h, but it increased again at 2 h to reach a maximum level, then decreased gradually (Figure 3b). On PT group, application of the FSS induced two peak levels of expression of FAK mRNA. First peak level was reached at 1 h under 8 dynes/cm^2^ FSS, and second peak level was at 4 h under 12 dynes/cm^2^ FSS (Figure 3(a, b)). Distinctly, at 16 dynes/cm^2^ FSS, first peak level of FAK was reached at 0.5 h, and the level at 12 dynes/cm^2^ was higher than at 8 dynes/cm^2^ (Figure 3c). The FAK mRNA expression of SLA group was activated. Exposure of 8 and 16 dynes/cm^2^, the FAK level increased and reached the maximum level at 2 h (Figure 3(a–c)). When exposed to 12 dynes/cm^2^, the FAK level reached maximum at 1 h, then it got down gradually.

### The FAK protein expression in different titanium implant

**Figure 4.**
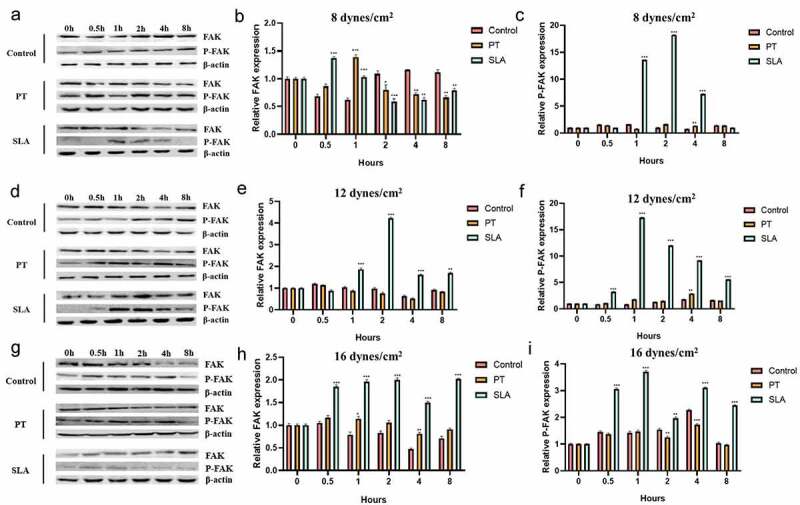
Effect of FSS on the FAK gene expression of MG63 on different titanium implant. Cells were exposed to FSS at 8 dynes/cm^2^ (a–c), 12 dynes/cm^2^ (d–f), 16 dynes/cm^2^ (g–i). The western blot results of FAK and P-FAK in cells exposed to FSS at 8 dynes/cm^2^ (a), 12 dynes/cm^2^ (d) and 16 dynes/cm^2^ (g). The proteins levels FAK were calculated in cells exposed to FSS at 8 dynes/cm^2^ (b), 12 dynes/cm^2^ (e) and 16 dynes/cm^2^ (h). The proteins levels P-FAK were calculated in cells exposed to FSS at 8 dynes/cm^2^ (c), 12 dynes/cm^2^ (f) and 16 dynes/cm^2^ (i). *P < 0.05, **P < 0.01, ***P < 0.001

Next, we explored the FAK protein expression in different groups by western blotting. Under the 8 dynes/cm^2^, the expression of FAK increased in 0.5 h in SLA group, then decreased gradually. The expression of p-FAK increased in 1, 2 and 4 h (Figure 4(a–c)). Under 12 and 16 dynes/cm^2,^ the expression of PAK was enhanced in 1, 2, 4 and 8 h, and p-PAK expression also augmented in 0.5, 1, 2, 4 and 8 h (Figure 4(d–i)).

## Discussion

MG-63 cells are derived from osteosarcomas, malignant bone tumors consisting of cells with abnormal cellular functions, and are commonly used for osteoblastic models as the cell synthesizes osteoid and exhibits increase alkaline phosphatase and osteocalcin hence provides a good cellular model for testing bone implant materials. Therefore, in the current study, MG-63 cells were recruited for the cell study. The morphology of an implant surface, such as microtopography and roughness, is considered to be an important element that affects the osseointegration of the implant. Some studies have indicated that implants with a coarse surface might be preferable compared with mechanically polished smooth titanium implants [24]. SLA is the most prevalent treatment for titanium implants and shows a significant positive effect on cells [25]. Therefore, the two representative titanium surfaces, PT titanium surface and SLA titanium surface, were chosen for the present study. The major findings of this study were that surface topography and the level and duration of FSS had effects on the cellular proliferation and the expression of FAK in MG-63 cells, suggesting that surface topography influences the effects of FSS on MG-63 cells on implant-bone interface and FAK may play a pivotal role in the mechanotransduction on the implant-bone interface.

Our results confirmed our previous findings that cells respond to shear stress with increased cell proliferation and the effects of FSS are dependent on the level of FSS and the duration of exposure [26–28]. Similarly, studies have proved that FSS has a marked effect on cells, in terms of cellular attachment, migration, morphology, proliferation, and differentiation [28,29]. In addition, the osteoclast cell migration direction can be influenced by the FSS magnitudes and gradients [30]. Under the FSS ranging from 12 to 14 dynes/cm^2^, regarding physiological level, the cellular biological behaviors occur significantly change in a short time, such as upregulation of the activity of autogeneration and expression of PEG2, NO, TGF-β and c-Fox [31]. The low level of FSS, even loading a long time, may result in little change of cell function and morphology [32,33]. The high level of FSS has a negative influence on the cell behaviors. Excessive duration of FSS contributes to the cell apoptosis, especially under the high level of FSS [34,35].

On the other hand, our results showed surface topography influenced the effects of FSS on MG-63 cells on implant–bone interface. One reason for the phenomenon is that surface morphology and roughness have effects on osteoblast behaviors, in terms of orientation, migration, adhesion, proliferation and differentiation [36–38]. Existing studies have shown that increasing surface roughness inhibited osteoblast proliferation but promoted cell differentiation [38,39]. Osteointegration is formed with contact osteogenesis and distant osteogenesis [40]. Osteoblast adhesion is essential to subsequent proliferation and differentiation [40]. Interestingly, the shallow hollows existing in the rough topography of SLA measured around 20–30 μm in diameter are equivalent or larger than the size of spread osteoblast cells or osteoblast-like cells. MG63 cells have been shown to adopt a three-dimensional (3D) shape when attaching inside the 30 μm diameter cavities, suggesting cell morphology is dependent on the presence of sub-micron-scale structural features. It is known that surface roughness is not only closely correlated to cellular attachment but also osteoblast activity [36,41]. In vivo and in vitro studies have demonstrated rough surfaces also positively affected cellular activity compared with machined titanium surfaces [41,42]. The evidence suggests the appreciable impact of titanium surface on cell proliferation or differentiation, cell adhesion and cellular activity. The differences in cell morphology and cellular activity may be the reason for the marked effect of FSS. Additionally, preclinical and clinical studies prove that surfaces with micro-scale and submicron scale roughness exhibit power bone–implant contact than smooth surfaces [43,44]. It suggested that a rough surface has an opposing effect on shearing force in the bone–implant surface [39].

But the mechanisms involved in the biological response are still not thoroughly understood. Various studies have already shown that the rough surface upregulates cellular adhesion and expression of ECM, including fibronectin (FN), type I collagen and osteopontin [45,46]. Recent evidence suggested that the increased focal adhesions (FAs) induced by rougher surfaces, may explain the sensitivity of osteoblasts to the surface features [47]. It implied that ECM–integrins–cytoskeleton pathways are involved in the regulation of the cellule behaviors.

Furthermore, as observed by confocal microscopy, shear stress causes the recruitment of signaling complexes to focal adhesions and cytoskeletal rearrangement. It has been assumed that FSS stimulates cells through integrins–cytoskeleton pathways. Interestingly, studies have shown that shear stress induces transient activation of FAK, which is located at focal adhesions [14]. FAK was correlated to FAs turnover, regulating the recombination of cytoskeleton and lamellipodia [14]. Studies have reported that shear stress induces lamellipodial protrusion, and recruits FAK to the new Fas formation under the lamellipodia in the flow direction [48]. FAK, as a key signal component at focal adhesions, is critical in FSS-stimulated signal transduction and directional migration of cells [48].

Secondly, the major finding of the present study is that FAs recruitment and actin cytoskeletal reconstruction stimulated by shear stress was also associated with the activation of small GTPases Rac and Rho [49]. With regard to membrane protrusion, Rac stimulates actin polymerization via the WAVE-Arp2/3 complex pathway so that induces membrane protrusion [49]. The phosphorylation of FAK results in the upregulation of Rac activity and downregulation of Rho activity, suggesting FAK can crosstalk with Rho GTPases-mediated pathways [49,50]. It has been observed that fibroblasts from FAK-/- mice failed to transiently inhibit Rho activity, whereas re-expression of FAK restored normal Rho regulation [51]. The relation between FAK and the small GTP-binding protein Rho is still unclear.

Consequently, the effect of FSS on MG-63 on implant-bone interface is correlated to surface roughness and topography, cell activity, cell attachment, cell morphology, and the level and duration of FSS. The roles of FAK in the mechanotransduction of FSS on MG-63 on implant–bone interface remains to be determined. In addition, the present cell experiments just focus on the effects of FSS on cell proliferation, its influence on cell differentiation should be further examined. The presents study only concentrated on the in vitro experiments, the in vivo experiments and clinical studies should be taken into consideration to verify the current findings.

## Conclusions

The present study demonstrated that different surface modifications created different microtopography of titanium plates. SLA surface contributes to the expression of FAK. Cell proliferation and FAK levels were stimulated by FSS and regulated by a marked synergistic effect of surface topography and the level and duration of FSS. In the future, the effects of FSS on FAK expression in human osteoblast-like cells on different surface modifications of Titanium should be further explored in animal experiments and clinical studies.

## Data Availability

The data that support the findings of this study are available from the corresponding author upon reasonable request.

## HIGHLIGHTS

1. SLA surface contributes to the expression of FAK.
2. Cell proliferation and FAK levels were stimulated by FSS.
3. Titanium surface regulates FSS’s influence on osteoblast proliferation.
